# Open-label versus double-blind placebo treatment in irritable bowel syndrome: study protocol for a randomized controlled trial

**DOI:** 10.1186/s13063-017-1964-x

**Published:** 2017-05-25

**Authors:** Sarah Ballou, Ted J. Kaptchuk, William Hirsch, Judy Nee, Johanna Iturrino, Kathryn T. Hall, John M. Kelley, Vivian Cheng, Irving Kirsch, Eric Jacobson, Lisa Conboy, Anthony Lembo, Roger B. Davis

**Affiliations:** 1 Department of Medicine, Division of Gastroenterology, Beth Israel Deaconess Medical Center, 330 Brookline Avenue, Boston, MA 02215 USA; 2 Program in Placebo Studies, Beth Israel Deaconess Medical Center/Harvard Medical School, 330 Brookline Avenue, Boston, MA 02215 USA; 3 Department of Global Health and Social Medicine Harvard Medical School, 641 Huntington Avenue, Boston, MA 02115 USA; 4 Division of Preventive Medicine, Brigham and Women’s Hospital/Harvard Medical School, 900 Commonwealth Avenue, Boston, MA 02215 USA; 5 Department of Psychology, Endicott College, 376 Hale Street, Beverly, MA 01915 USA; 6 Department of Medicine, Division of General Medicine and Primary Care, Beth Israel Deaconess Medical Center, 330 Brookline Avenue, Boston, MA 02215 USA

**Keywords:** Placebo effects, Open-label placebo, Irritable bowel syndrome, Research methods, Multi-disciplinary models, Peppermint oil

## Abstract

**Background:**

Placebo medications, by definition, are composed of inactive ingredients that have no physiological effect on symptoms. Nonetheless, administration of placebo in randomized controlled trials (RCTs) and in clinical settings has been demonstrated to have significant impact on many physical and psychological complaints. Until recently, conventional wisdom has suggested that patients must believe that placebo pills actually contain (or, at least, might possibly contain) active medication in order to elicit a response to placebo. However, several recent RCTs, including patients with irritable bowel syndrome (IBS), chronic low back pain, and episodic migraine, have demonstrated that individuals receiving open-label placebo (OLP) can still experience symptomatic improvement and benefit from honestly described placebo treatment.

**Methods and design:**

This paper describes an innovative multidisciplinary trial design (*n* = 280) that attempts to replicate and expand upon an earlier IBS OLP study. The current study will compare OLP to double-blind placebo (DBP) administration which is made possible by including a nested, double-blind RCT comparing DBP and peppermint oil. The study also examines possible genetic and psychological predictors of OLP and seeks to better understand participants’ experiences with OLP and DBP through a series of extensive interviews with a randomly selected subgroup.

**Discussion:**

OLP treatment is a novel strategy for ethically harnessing placebo effects. It has potential to re-frame theories of placebo and to influence how physicians can optimize watch-and-wait strategies for common, subjective symptoms. The current study aims to dramatically expand what we know about OLP by comparing, for the first time, OLP and DBP administration. Adopting a unique, multidisciplinary approach, the study also explores genetic, psychological and experiential dimensions of OLP. The paper ends with an extensive discussion of the “culture” of the trial as well as potential mechanisms of OLP and ethical implications.

**Trial registration:**

ClinicalTrials.gov, identifier: NCT02802241. Registered on 14 June 2016.

**Electronic supplementary material:**

The online version of this article (doi:10.1186/s13063-017-1964-x) contains supplementary material, which is available to authorized users.

## Background

Placebo pills are typically comprised of inactive ingredients, such as lactose or microcrystalline cellulose, and are designed to match active pharmaceuticals in appearance without having any physiological effects on symptoms. Though designed specifically to be inactive and physiologically ineffective, rigorous evidence has demonstrated that treatment with placebo can produce effects beyond that which one would expect from spontaneous improvement or natural waxing and waning of symptoms [1]. These so-called “placebo effects” are believed to represent relief of symptoms in the context of the therapeutic encounter, complete with its symbols (e.g., white coats), rituals (e.g., taking pills), expectancies (e.g., “medication can make me feel better”), hope (e.g., “there are still possibilities”), and interactions (e.g., therapeutic relationship). Furthermore, recent evidence has begun to delineate a specific and quantifiable neurobiology associated with response to placebo [2, 3].

Although placebo is widely used in clinical and research settings, until recently it has always been administered “concealed” in the context of double-blind randomized controlled trials (RCTs) where patients are aware of the likelihood that the prescribed treatment may or may not contain an active ingredient or may be presented in an ethically dubious deceptive manner in which patients are unaware that the prescribed pills contain either an inactive ingredient or an active substance that had no effect on their condition [4]. Here, we will briefly review the academic literature on the traditional use of blinded placebos, discuss recent research using open-label placebo (OLP), and describe the methodology and rationale for our current RCT, which was designed to compare open-label and double-blind placebo (DBP) in a sample of participants with irritable bowel syndrome (IBS). We also discuss the possible mechanisms of OLP and the ethical implications.

### Traditional use of placebo in research and clinical practice

To date, hundreds of thousands of research participants have received DBPs in the context of RCTs. In an RCT, the placebo group serves to control for placebo responses and for other common factors that may result in symptomatic improvement such as detection biases, natural waxing and waning of symptoms, and regression to the mean. If the experimental treatment is found to result in more symptomatic improvement when compared to the placebo treatment, the observed differences are generally considered to be due to the active drug ingredient and not to incidental factors such as placebo effects or spontaneous improvement. In most cases, administration of placebo to research participants occurs after providing informed consent, in which it is clearly explained that there is a chance of receiving either the active study intervention or an inactive placebo intervention. As such, even though research participants are kept blinded to their treatment assignments, there are relatively few ethical concerns regarding placebos in RCTs [5, 6].

The utilization of placebo in clinical practice, however, is traditionally much less transparent and is associated with a long history of ethically questionable practice [7, 8]. After the widespread adoption of the RCT as the “gold standard” of evidence-based research in the 1960s and the acknowledgement of patient rights in medicine, prevailing ethics renounced the deceptive use of placebos in clinical practice [9–11]. Nonetheless, without much public discussion, deceptive use of placebos continues to be commonly used in clinical settings. For example, a national randomized survey published in 2008 of 679 practicing internists and rheumatologists in the USA found that approximately 50% of respondents regularly prescribed placebos [4] and a survey of 208 randomly selected general practitioners in Germany found that 76% reported prescribing placebos [12]. Systematic reviews of such research have found similar or even higher rates of placebo utilization around the world [13]. This line of research has also consistently found that “impure” placebos – i.e., genuine medications that physicians understood would have no intrinsic pharmacological action on patients’ symptoms – were much more commonly prescribed than “pure” placebos such as sugar pills [14]. In these surveys, physicians clearly indicated that placebos were prescribed for “psychological benefits,” not uncommonly as part of a “watch-and-wait” strategy, and that patients rarely, if ever, were told that they were receiving placebos or pharmaceuticals serving the purpose of placebos. The reason that providers do not inform their patients of their choice to prescribe placebo likely stems from the conventional wisdom that patients must believe that placebo pills are “real” medications and that informing patients that they have been prescribed a placebo would “erase” the placebo effect; in other words, it is widely assumed that honesty and transparency are incompatible with placebo effects [15].

### Open-label placebo

Can placebo effects be ethically harnessed with honestly prescribed OLP? Until recently this question was considered absurd. However, there is significant value in exploring whether an honest placebo strategy might be helpful in treating common symptoms, especially in “watch-and-wait” treatment strategies or before prescribing medications with potentially serious side effects. Recently, there have been several proof-of-concept studies testing whether OLP can improve clinical outcomes compared to a treatment-as-usual (“no-treatment control” (NTC)) group. The first such study involved a sample of patients with IBS (*n* = 80) randomized to OLP versus NTC [16]. Among OLP patients, 60% reported “adequate relief” of their IBS symptoms versus 37% “adequate relief” in NTC patients (*p* = 0.03). A more recent study randomly assigned patients with chronic low back pain (cLBP; *n* = 83) to a similar design and reported that, on a 0-to-10 composite pain scale, patients receiving OLP reported 30% reduction of usual and maximum pain compared to reductions of 9% and 16% in usual and maximum pain, respectively, in the continued NTC (*p* < 0.001). There was a 29% reduction in pain-related disability in patients receiving OLP compared to 0.02% in the continued NTC [17]. A third, elaborate, within-subject randomized experiment with patients serving as their own control during acute episodic migraine attacks (*n* = 459 documented attacks in 66 patients) included a nested comparison of OLP versus NTC and found that the OLP reduced pain by 15% compared to 15% worsening in the NTC, so that the total mean difference between conditions was 30 percentage points, *p* = 0.001) [18]. Other similar, but much smaller, studies in allergic rhinitis [19] and depression [20], as well as other somewhat similar studies using placebo-conditioning models in pediatric attention-deficit hyperactive disorder [21], insomnia [22], psoriasis [23], and healthy participants [24], have reported similar, positive outcomes. Clearly, many questions remain regarding whether OLP can be adopted for clinical use. We have recently begun a National Institutes of Health (NIH)-funded RCT in an attempt to answer some of these unresolved questions.

### The current study

This paper describes the methodology of an ongoing RCT, titled “Efficacy of open-label vs. double-blind treatment in IBS,” designed specifically to address the OLP research areas outlined below. This project brings together a multidisciplinary team of clinical, behavioral, and basic scientists to explore a series of rarely examined questions in OLP.

### Primary areas of interest

**Fig. 1:**
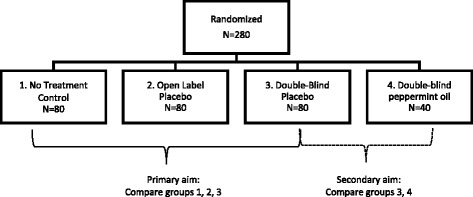
Treatment arms and sample sizes

The primary aim of this study is to compare symptoms after 6 weeks of OLP, DBP, and NTC in a sample of individuals with IBS (Fig. 1). In order to provide DBP treatment, this study includes a nested trial comparing double-blind peppermint oil and DBP. Peppermint oil was chosen for use in the double-blind arm because of its suggested antispasmodic effects on the gastrointestinal tract [25, 26].

#### Replication and expansion of previous findings

Although the existing findings in OLP research are provocative, it is not uncommon for preliminary research studies to report compelling findings that fail to replicate. Therefore, it is our aim to clarify (1) whether current findings on OLP can be replicated and (2) under what circumstances they are most likely to be replicated. The present study is an attempt to replicate and expand upon the first RCT of OLP that was performed in patients with IBS [16], in which we compared OLP with NTC and found significantly more improvement in OLP compared to NTC. In the current study, we will compare OLP, NTC, and DBP and will collect data from a larger sample over a longer period of time, increasing the sample size from 80 to 280 participants and the treatment time from 3 to 6 weeks. An increased treatment time will allow us to evaluate the duration of the OLP effect as most studies to date have administered OLP for shorter durations and there are not yet any existing data to clarify for how long taking OLP might work.

#### Comparing OLP, DBP, and NTC

Clearly, OLP and DBP conditions are inherently different in that DBP groups have knowledge that they *could* be receiving active treatment, while OLP groups do not. To our knowledge there is no information on how OLP and DBP compare in modifying outcomes. This study seeks to identify whether there are smaller, similar, larger, or highly variable effects when comparing one to the other and to compare both to NTC.

There are several potential implications that result from these research questions. First, this information could help to identify which symptoms or syndromes should be investigated with OLP next. Assuming that objective pathophysiology is not easily changed by placebo interventions [1, 27], there are likely certain symptoms that will respond better to OLP and some that will not respond at all. For example, the placebo response in DBP RCTs (which includes spontaneous improvement and regression to the mean due to the lack of an NTC group in most RTCs) is large for the symptoms of benign prostatic hyperplasia [28], menopause-related or cancer-related hot flashes [29], and fatigue [30]. If we could determine the relationship between OLP and DBP in at least one condition that is primarily based on patient-reported symptoms, we may be able to estimate the effects of OLP on other chronic, functional illnesses and could begin to prioritize worthy targets of future RCTs testing OLP treatments. Finally, understanding how OLP and DBP compare will allow us to begin to evaluate whether there are different mechanisms in a DBP response versus an OLP response and whether there might be different psychological or neurobiological profiles to predict each response.

### Secondary areas of interest

Our secondary areas of interest are largely informed by our previous placebo studies in IBS:

#### Genetic predictors of placebo response

The search for genetic biomarkers for placebo response has the potential to advance clinical care (e.g., titrating medication doses) and clinical trial design (e.g., enrichment strategies). In our previous large RCT (*n* = 262), testing components of placebo effects in IBS [31], we found that the number of methionine alleles in the catechol-*O*-methyltransferase (COMT) *val158met* polymorphism was associated with placebo response, especially when placebo was combined with a supportive patient-provider relationship [32]. Due to expense and sample size, we were unable to undertake a genome-wide association study (GWAS). Instead, we hypothesized that, since dopamine release in the brain is implicated in placebo responses [33, 34], genetic variation in COMT, an enzyme that degrades catecholamines such as dopamine, might be associated with placebo response. Using a candidate genetic analysis in our RCT of placebo treatment in IBS, we demonstrated that the low-activity allele (met/met) of the COMT *rs4680* polymorphism, known to result in higher levels of dopamine, was indeed associated with an increase in placebo response. In the present study, we plan to see if COMT also modifies response to OLP. Since our identification of COMT several other genes have been implicated in modification of the placebo response [35]. In this study we will conduct a candidate genetic analysis of genes hypothesized to be associated with placebo response, collectively known as the placebome.

#### Psychological predictors

In our previous large RCT testing components of placebo effects in IBS [31], we found that higher levels of extraversion, agreeableness, and openness to experience are associated with increased response to placebo treatment, in the context of an augmented patient-practitioner relationship [36]. We will use a series of well-validated psychological measures to test whether this is also true in OLP.

#### Qualitative interviews

In our team’s previous large RCT testing placebo effects in IBS [31] we included an embedded qualitative study that produced four groundbreaking qualitative papers demonstrating a large gap between what patients think, feel, and consider important compared to what researchers and physicians measure and believe [37–40]. In the current study, we have adopted this same method in order to collect the first in-depth interviews ever performed on patients receiving OLP.

#### Nested peppermint oil study

In order to provide DBP treatment, our current study includes a nested trial comparing double-blind peppermint oil and DBP. Peppermint oil was chosen for use in the double-blind arm because of its suggested antispasmodic or “soothing” effects on the gastrointestinal tract [25, 26]. Based on five RCTs identified by the American College of Gastroenterology Task Force in 2014, the number needed to treat with peppermint oil is an impressive three (95% CI 2 to 4) [41]. While promising, most of the identified studies were of low quality with significant methodological limitations and none of the trials were conducted in the USA. As a result, use of peppermint oil for IBS is limited in clinical practice and a better understanding of the efficacy of peppermint oil in treating IBS will have high clinical utility.

## Methods/design

**Fig. 2:**
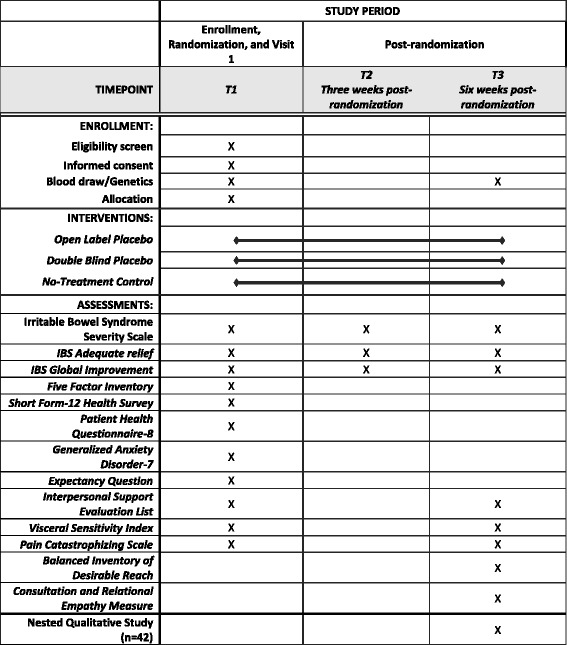
Schedule of enrollment, interventions, and assessments

Below we describe the specific methods used to test whether there are differences in outcomes between OLP treatment, DBP treatment, and NTC. Recruitment began in July, 2016 and is expected to be complete in 2019 yielding a sample of 280 participants randomized to four arms. See Fig. 2 for a detailed timeline of the study which accords with the Standard Protocol Items: Recommendations for Interventional Trials (SPIRIT) figure (Additional file 1).

### Subject selection

#### Inclusion/exclusion criteria

Detailed inclusion and exclusion criteria are listed in Table 1. We diagnose IBS based on physician interview using the Rome IV criteria published in 2016 which has demonstrated good specificity and sensitivity for IBS [42]. The Rome IV criteria require recurrent abdominal pain, starting at least 6 months previously, and occurring at least 1 day/week in the last 3 months, associated with at least two of the following three features: [1] related to defecation; [2] associated with change in frequency of stool; and [3] associated with a change in form (consistency) of stool. Participants who meet criteria for IBS and who report at least moderate symptom severity at baseline visit (determined by a score of >175 on the Irritable Bowel Severity Scoring System) [43] are eligible for the study.

**Table 1:** Inclusion and exclusion criteria used to determine eligibility for participation

Inclusion
1. Provide signed and dated informed consent and understand the nature of the study sufficiently to allow completion of all study assessments
2. Be ambulatory, community dwelling, 18 to 80 years, inclusive
3. Meet Rome IV diagnostic criteria for IBS
4. Have IBS of at least moderate severity, i.e., have a score on the IBS-SSS of >175 (0–500) at the baseline visit*
5. If the patient is on medications which affect the gastrointestinal tract or visceral sensation (e.g., tricyclic antidepressants, fiber, antispasmodics, etc.), they must be on a stable dose for at least 1 month prior to entering the study and for the duration of the study*
*Participants will be allowed to rescreen once if they do not meet inclusion criteria #4 or #5 at the initial screening visit
Exclusion
1. Self-reported pregnancy or planned pregnancy within the next 2 months
2. Have an established diagnosis of any concomitant bowel disturbance that would interfere with the assessment of efficacy or safety in the study (e.g., Hirschsprung’s disease, diverticulitis, colonic ischemia)
3. Report warning symptoms (e.g., rectal bleeding, weight loss >10%, iron deficiency anemia, etc.) otherwise not explained
4. Have undergone previous abdominal surgery to the intestines (with the exception of uncomplicated appendectomy, cholecystectomy, hysterectomy, or polypectomy >6 months prior to enrollment)
5. Have a history of drug, excluding nicotine or caffeine, or alcohol abuse within 2 years of entry into the study
6. Exhibit abnormalities on physical examination, unless judged to be clinically insignificant by the investigator. Such cases will be noted
7. Current, within the past 30 days, therapeutic use of enteric-coated peppermint oil for the treatment of IBS
8. Known or suspected peppermint or soybean oil allergy
9. Severe acid reflux (>3 episodes of heartburn or regurgitation per day on average over a week)
10. Inability to speak or read English
11. Unable or unwilling to cooperate with the study protocol or considered by the investigator to be unsuitable for the study

*IBS* irritable bowel syndrome, *IBS-SSS* Irritable Bowel Syndrome Severity Scoring System

Participants are allowed to continue any IBS medications as long as they have been on stable doses for at least 30 days prior to entering the study and agree not to change medications or dosages during the trial. Any nonpharmacological treatments for IBS (e.g., meditation, dietary regime, etc.) are also allowed as long as they are on a stable pattern/behavior for at least 30 days prior to entering the study.

### Study interventions

The study involves four groups as follows: (1) no-treatment control (NTC) (*n* ≈ 80); (2) open-label placebo (OLP) (*n* ≈ 80); (3) double-blind placebo (DBP) (*n* ≈ 80); and (4) double-blind peppermint oil (*n* ≈ 40). See Fig. 1. Because the nested peppermint oil comparison is a secondary aim, arising from necessary inclusion of peppermint oil in order to test DBP, we chose to randomize fewer participants into this arm in order to increase power for the primary comparison of OLP, DBP, and NTC.

#### The pills: placebo and peppermint oil softgels

Three of the four study groups are administered either a placebo (*n* = 160) or peppermint-oil softgel (*n* = 40). The OLP and DBP pills are identical to each other and both are matched to the peppermint oil. In the peppermint oil group, 0.2 mL-dose peppermint oil softgels are used (Pepogest™; Boca Raton, FL, USA). In the placebo groups, treatment is supplied as 0.2 mL soybean oil in order to match the peppermint oil softgels in appearance (manufactured by SoftGel Technologies Inc; Los Angeles, CA, USA). Participants in the peppermint oil and placebo groups receive identical instructions to take one softgel three times per day, approximately 30 min before meal times. The bottle of pills is labeled as “Open Label Placebo Capsule” in the OLP arm and as “Placebo or Peppermint Oil Capsule” in the double-blind arm.

#### The setting: interactions with clinicians and study staff

We notify potential participants about the study via (1) mailed postcards, (2) flyers posted in greater Boston, and (3) physician referrals. Participants are first introduced to the study (in person or by phone) by trained study staff. This initial interaction provides a transparent description of the aims of the study and outlines the four groups into which participants might be enrolled. During this first encounter, OLP is introduced as a “novel, mind-body intervention” and the rationale for the study is briefly discussed.

During study visits, clinicians are instructed to be warm, empathic, natural, and truthful about the design and methods of the study with all patients. Although patients are informed of the design of the study during the first contact with the recruiters, physicians give all patients a detailed overview of the study during visit 1. After introducing the study and answering any questions, the physician opens a randomization envelope and informs patients of their assignment to either the OLP group, the double-blind group, or the NTC group. In the OLP group, the physician is aware of assignment, as they would be in clinical practice. Just as in a standard double-blind RCT, neither the participants or the study staff are aware of which blinded group (peppermint oil or placebo) is assigned in the double-blind condition. After randomization, participants are given a semiscripted description of their group assignment (Table 2). This semiscripted interaction lasts approximately 20–25 min (including informed consent) for all patients in all study groups. The script for the OLP group is similar to our previous OLP study and the script for the double-blind and NTC are similar to what would happen in a typical RCT. The physician makes every effort to assure equal time, attention, encouragement, and patient-physician relationship for every arm of the study. The study time with research staff/physicians totals approximately 1 h. Including the time needed to complete study questionnaires the entire first visit takes approximately 1.5 h.

**Table 2:** Core components of scripted interactions between study clinicians and study participants in each arm

Open-label placebo
Interaction is geared toward making participants comfortable taking open-label placebo and reducing negative feelings about placebo. Specifically, patients will be told the following four points:
1. In clinical trials, IBS patients treated with placebos often improve nearly as much as patients on active medication
2. In order to reduce concern that that any placebo effect a participant might experience is “all in the head,” classical conditioning (“like Pavlov’s dogs”) will be suggested as a possible mechanism to explain how such self-healing can happen automatically
3. To suspend disbelief and encourage an open mind about the effectiveness of the treatment, patients will be told that positive expectations may be helpful, but it is normal to have doubts about open-label placebo. We can understand why patients might think the study is unusual
4. To encourage adherence, patients will be told that it is most important to take the pills faithfully. Research shows that patients who are more adherent – even to placebo – improve more than those who are less adherent
5. Participants are provided with reassurance that they may not notice changes right away. They are informed that some people respond to placebo “gradually” and others respond “quickly” and that this varies across individuals
Double-blind placebo and double-blind peppermint oil
1. Provide rationale for doing a double-blind experiment and the principle of equipoise
2. Provide a rationale for why peppermint oil might be effective: “Peppermint oil has been used for many years to ‘soothe’ the GI tract and is known to reduce contractions or spasms in the GI tract”
3. Explain that peppermint oil in IBS has never been tested in the USA
4. Provide a rationale for why placebo might be effective: “In early clinical trials IBS patients treated with placebos often improve nearly as much as patients on active medication”
5. Participants are provided with reassurance that they may not notice changes right away. They are informed that some people respond to placebo “gradually” and others respond “quickly” and that this varies across individuals
No-treatment control
1*.* Explain the importance of this research to develop effective treatments for IBS
2. Explain that it is critical to measure the normal waxing and waning of IBS symptoms in the no-treatment control group in order to understand the placebo and peppermint effects detected in the other arms of the study
3. Highlight the importance of not changing anything that they are regularly doing for the IBS during the trial
4. Explain that the study physician will provide some specific suggestions and options at the end of the trial

*GI* gastrointestinal, *IBS* irritable bowel syndrome

At each follow-up visit, patients in OLP and DBP arms are asked to bring in any remaining pills that they may have so that a blinded assessor can count the pills in order to track compliance.

### Culture of the trial

The general context and ambiance of a pharmaceutical RCT is not usually described in protocols. Most researchers assume that these details are less important than the nuts and bolts of the implementation. Given that placebo effects are ultimately the result of the entire context of a clinical interaction we want to share some of the following description of the culture of the current trial.

First, we believe that absolute honesty is critical. Placebos still conjure the idea of deception. In all interactions and discussions with participants, our team tries to be comfortably transparent. Participants may be suspicious of placebo research and we want to avoid this whenever possible. Second, we do not pressure participants to believe or expect that open-label treatment will work. Obviously, we would not be doing a RCT if we thought it was absolutely certain, and more importantly, our team has its own doubts (regardless of our earlier study). Research suggests that patients are rational and are commonly uncertain of their expected outcomes of participation in trials [37]. Importantly, our hospital is a tertiary facility and our patients have usually seen multiple physicians for IBS who have given them positive expectations that have been false. They have undergone many episodes of therapeutic failure. Therefore, we do not suppress or deny uncertainty. And no pressure is ever applied to patient’s experience. Our motto about taking placebos could be described as: “let what happens, happen” and we often say this to patients.

Another specific contextual point unique to this OLP study is that it has both OLP and a potential active drug (peppermint oil). In our experience, patients are not unlike health care providers in that when offered a choice, they want a “real” drug. Like society at large, there is a prevailing belief that compared to a drug, placebos are a sort of consolation prize or, more colloquially, the “booby prize.” In our advertisements, we speak of a novel-mind body intervention that harnesses placebo effects. We want patients to be open to placebo effects and we try to avoid the possibility that initially advertising peppermint oil will make people think that the placebo is the less important intervention. When potential participants call, we explain the entire trial transparently and we clearly explain that we include peppermint in the trial in order to compare OLP and DBP and because preliminary results suggest that it may be beneficial for IBS. During the intake, we make it clear that evidence exists that peppermint oil may be helpful.

As will be discussed below, the mechanisms of OLP are not yet understood. Given this, our trial emphasizes that behaving naturally, respectfully and considerately is critical. Following what we tell our patients, our team assumes that the active ingredient is “automatic” (or at least very hard to describe scientifically or psychologically at this point) and involves the sum and/or interaction of many factors, including the words, nonverbal communication, symbols, rituals, and behaviors involved from the first advertisement notice to the screening call to the intake procedures and all visits.

### Study timeline

In all of the study groups, participants attend three in-person visits over the course of 6 weeks. In study visit 1, participants provide informed consent, complete questionnaires, meet with a study clinician, and are randomized into study groups. In visit 2 (3 weeks post randomization) and visit 3 (6 weeks post randomization), participants complete questionnaires and meet briefly with a study clinician. Visits 1 and 3 also include a blood draw (20 mL each visit), see section titled “Genetic screening” below for additional details.

Additionally, 42 patients will be randomly selected to participate in the nested qualitative study and will undergo a detailed interview with a medical anthropologist or sociologist upon completion of their final study visit.

### Measures

Measures were chosen to increase our understanding of the placebo phenomena in this population. All measures are outlined in Table 3. When feasible, we selected continuous rather than dichotomous measures. Our preference for continuous scales is supported by a meta-analysis that compared placebo arms with wait-list controls, and found that studies using continuous variables were more likely to detect significant placebo effects [44].

**Table 3:** Measures to evaluate symptoms, quality of life, and patient expectancy

Scale	Description
Primary outcome measure	
Irritable Bowel Symptom Severity Scale (IBS-SSS)	This 5-item questionnaire provides a simple way to scale IBS symptoms and the progress of the disease. Items consider pain, distension, bowel dysfunction, and quality of life/global wellbeing. Scores show good reliability and sensitivity to change
Secondary outcome measures	
Symptom evaluation	
IBS-Adequate Relief	A single yes/no question makes up this simple global measure of symptom relief in the past week
IBS-Global Improvement	A single question provides a measure of improvement in the past week using a 7-point scale
Daily Symptom Diary	Questions regarding subject symptoms will be completed daily for the 7-day period prior to visit 2 and visit 3
Quality of life	
Short Form-12 Health Survey (SF-12)	This 12-item scale measures emotional and physical health and wellbeing
Psychosocial	
Patient Health Questionnaire-8 (PHQ-8)	An 8-item scale that is used as a diagnostic and severity measure for depressive disorders in clinical studies
Generalized Anxiety Disorder-7 (GAD-7)	A 7-item scale that is used as a diagnostic and severity measure for anxiety disorders in clinical practice and research
Visceral Sensitivity Index	A 15-item measure of gastrointestinal symptom-specific anxiety
Pain Catastrophizing Scale	A scale that measures the tendency to magnify or exaggerate the seriousness of pain sensations
Five Factor Inventory	A 60-item instrument that measures the “Big Five” dimensions of personality
Interpersonal Support Evaluation List	Designed to measure perceptions of social support among individuals in the general population
Balanced Inventory of Desirable Reach	Describes the tendency of respondents to answer questions that will be viewed favorably by others
Patient experience	
Consultation and Relational Empathy Measure	Assesses physician empathy and relational skills on 10 ordinal items, which are then summed
Expectancy Question	“If I receive placebo/peppermint oil/no additional treatment, I expect my IBS symptoms to be: “(numerical rating scale running from zero “not improved at all” to 100 “completely improved”)

### Genetic screening

Genomic DNA will be extracted from whole blood drawn at visits 1 and 3 using Qiagen Blood kit (Valencia, CA, USA) following the manufacturer's protocol. TaqMan single nucleotide polymorphism (SNP) genotyping assays will be purchased from Applied Biosystems, (Foster City, CA, USA). Each visit will test for “placebome markers,” described in detail elsewhere [35]. Exploratory analyses will be performed for: the mu-opioid receptor polymorphism (*OPRM1 A118G*), fatty acid amide hydrolase (*FAAH*) polymorphisms *Pro129Thr*, monoamine oxidase gene polymorphisms, and serotonin-related polymorphism *CGTTLPR* and *G-703 T* (polymorphism in the tryptophan hydroxylase-2 (*TPH-2*) gene promoter). COMT and other candidate genotypes will be correlated with response to placebo or active drug treatment. Furthermore, potential correlations of the COMT and other genotypes and patient-disease characteristics, for instance pain experience at baseline, will be examined.

### Randomization, stratification, and blinding

All outcome assessments, at all study points, are performed by blinded research assistants. Patients on OLP are obviously not blinded; patients assigned to DPB or peppermint oil are told that they are in a regular double-blind RCT and are unaware of exact treatment assignment. Patients on the NTC are aware of assignment.

Randomized treatment assignments were generated by a program written by our biostatistician using SAS statistical software (SAS Institute, Inc., Cary, NC, USA). Treatment assignments are generated using permuted block randomization with randomly varying block sizes. Randomization is done in a 2:2:2:1 ratio (no-treatment control; open-label placebo; double-blind placebo; double-blind peppermint oil) and assignments to one of the four groups are sealed in sequentially numbered opaque envelopes.

Stratification is based on IBS symptom severity score (<300 and >300) and gender, resulting in four strata. Each stratum has a different color randomization envelope and a unique set of randomization ID numbers.

### Statistical considerations

Data are recorded and stored via Research Electronic Data Capture (REDCap) as the study is in progress. REDCap is a secure web interface with data checks during data entry and uploading to ensure data quality, and housed on secure severs.

#### Power and sample size

To calculate power for our primary analyses, we used our previous pilot trial testing OLP in IBS [16]. In that study, the effect size for the difference between OLP and no-treatment on the IBS Symptom Severity Scale was *d* = 0.53. For this study, there will be a total of 280 participants enrolled with 80 participants in the OLP, DBP, and NTC and 40 participants in the double-blind peppermint oil group. Using analysis of covariance (ANCOVA) to control for baseline scores leaves the power for the three-group comparison at 0.94. The comparison of peppermint oil and DBP using an ANCOVA approach provides power of 0.75 to detect an effect size of 0.60, an effect consistent with prior studies of peppermint oil.

#### Analyses

For our primary analyses, we will use a modified intention-to-treat analysis including any patient who was randomized and provided a baseline assessment and at least one post-baseline primary outcome assessment.

To test our primary aim (to determine whether 6 weeks of OLP, DBP, and no-treatment results in different clinical outcomes) we will conduct a one-way analysis of covariance. The covariates in the model will be the baseline IBS-SSS score, and gender. The covariates for the ANCOVA model will include the baseline value of the outcome measure as well as the two randomization stratification factors: symptom severity and gender. Assuming that there is a significant difference between the three groups, Dunnett’s analysis will be used to do pairwise analyses to evaluate specific differences between the groups.

For our secondary aims, we will use the following analyses: (1) analysis of covariance will be used to determine if double-blind peppermint oil results in greater improvement than DBP in patients with IBS; (2) exploratory analysis and correlation will be used to confirm and expand upon our previous findings regarding a genetic biomarker for the placebo response; (3) multiple regression will be used to test the association between personality traits and placebo outcome after controlling for baseline characteristics.

#### Missing data

Missing data minimization strategies include patient-retention efforts and a modified intent-to-treat analysis. Each individual patient-reported assessment will be captured electronically at each visit with missing responses prohibited by the electronic system. The modified intention-to-treat analysis will replace missing visit-3 outcome measures with visit-2 results, a last observation carried forward (LOCF) approach. If more than a few participants have missing visit-3 outcomes, we will conduct a sensitivity analysis using multiple imputation procedures to produce a full intention-to-treat analysis.

## Discussion

This paper describes the methodology of an ongoing, NIH-funded RCT designed to replicate and expand upon existing OLP research in patients with IBS. Until recently, directly harnessing the potential effects of placebo pills in an ethical and transparent manner that respected patients’ autonomy had rarely been considered. Clinicians and researchers have traditionally assumed honesty to be incompatible with placebo and have built clinical treatment and research protocols around the belief that the placebo effect would be eliminated if a patient were aware that a pill did not contain an active drug. OLP offers a possibility for directly and ethically harnessing placebo effects, especially in common, subjective, self-reported symptoms (e.g., chronic pain, functional abdominal symptoms).

The ongoing RCT described in this paper attempts to replicate earlier trials of OLP using a larger sample and longer treatment duration. Importantly, the current study will provide unique information about how DBP administration compares to OLP and will allow some very tentative inferences on what symptoms or syndromes should be targeted in future OLP trials. Furthermore, the secondary aims (both quantitative and qualitative) of this study will provide valuable information regarding the role of genetics and psychology in the OLP placebo response, which will also help further refine OLP treatment.

### Mechanisms of open-label placebo

One key concern regarding OLP is that it seems mechanistically implausible. How could patients who are knowingly receiving placebo still experience clinical benefit? Ultimately, we do not know the answer to this question and, clearly, more mechanistic research is needed. Our team holds many different perspectives on the issue and we agree to disagree until more data are accumulated. This said, some tentative discussion of neurobiological and psychological mechanisms may be helpful given the highly novel nature of the trial and research question.

### Neurobiology

The neurobiological mechanisms of placebo effects have begun to be well described for placebo administered under double-blind or deceptive conditions [2, 3]. As mentioned earlier, neurotransmitters are activated and specific, quantifiable, and relevant areas of the brain are engaged during traditional, blinded placebo administration. We assume that similar mechanisms are involved with OLP, but obviously do not know for certain. To our knowledge, the only basic scientific study of OLP examined healthy participants who were administered OLP for pain while undergoing functional magnetic resonance imaging (fMRI). The study followed a commonly used conditioning paradigm to elicit placebo effects and found that OLP administration engaged similar brain regions as DPB, except without prefrontal activation, suggesting that placebo analgesia may bypass areas of conscious expectation [24, 45]. Further research needs to be done in this area.

### Psychology

Currently, the two most prevalent psychological models to understand placebo effects are based on theories of expectancy and conditioning. Expectancy is usually thought of as a consciously accessible belief in the effectiveness of a therapy [46], while conditioning posits that previous experience taking (and benefitting from) effective medication (unconditioned stimulus) conditions an individual to experience benefit (conditioned response) in response to taking a pill (conditioned stimulus) [47]. In addition, conditioning and expectancy theories are widely considered complementary [48], rather than competing, in that conditioning experiences can shape expectancies. These theories are critical in placebo studies, but an exclusive reliance on these two theories may be insufficient for understanding OLP [49] given that it assumes that our patients had repeated positive experience with their IBS treatments or medicine in general. In fact, almost all of our patients in all our previous IBS RCTs were refractory and generally had unsuccessfully seen many gastroenterologists for their condition [37]. Therefore, our team has developed several additional hypotheses for the psychological mechanisms behind OLP.

### Expectancy

Expectancy remains the dominant psychological model in placebo research. It is often defined as explicit and accessible thoughts or expectations about probable outcomes in any given situation [46]. For example, when a doctor prescribes a medication, it is reasonable to expect that the medication will help to relieve one’s symptoms. What kind of expectancy, then, will patients have if told that they are receiving a placebo pill instead of an active medication? Perhaps not surprisingly, when we asked participants this exact question during exit interviews in our earlier OLP IBS study, most patients claimed that they had no, or only minor expectancy for, improvement [37]; and a recent OLP study in cLBP reported similar findings [17]. Nevertheless, both studies found OLP to be effective in improving symptoms. Although this general lack of positive expectancy combined with overall symptomatic improvement in both studies might suggest that expectancy is not a factor in OLP response, our team continues to entertain the possibility that expectancy may play an important role in OLP and that, perhaps, expectancy is not a simple or monolithic psychological entity. For example, it has been demonstrated that people can hold contradictory expectations simultaneously [50] and cognitive neuroscience has begun to parse “looking into the future” into more refined components such as prediction, preparation, anticipation, prospection, and expectation [51]. Several research studies have also suggested that placebos may work through nonconscious dimensions of expectancy, [52–54] which would suggest that our participants may experience a form of expectancy that is outside of their conscious awareness.

### Hope

The construct of “hope” may more accurately characterize some aspects of expectancy in placebo treatment or be an independent dimension. As mentioned earlier, in our previous study evaluating components of placebo effect in IBS [31], we included a substudy in which anthropologists performed in-depth interviews about patient’s experience with placebo. To our knowledge this is the first time that patients in a RCT who had undergone treatment with placebos have ever been asked to talk about their experiences in the trial. When queried about their expectations, patients almost uniformly denied that positive expectations influenced their decision to join the study. Instead, patients spoke of hope as what they needed to “get out of bed” every morning. Several other studies, with much larger samples, have replicated this finding that clinical patients describe hope, but not positive expectations, as one reason for entering a clinical trial [55, 56] .

Understanding what patients mean by hope is difficult even within these studies. In fact, a systematic review of the academic literature produces multiple theories or definitions of hope and a handful of distinct validated measures [57]. It seems that hope is a paradoxical combination of opposites, balancing despair and the counterfactual notion that things can improve – a kind of “tragic optimism” [58]. Hope (like empathy, compassion, envy, and shame) is clearly a complex emotion that involves deep feelings, expectations, cognitive reflection, and cultural rules of what is rational and reasonable when one looks into the future. This conceptualization of hope calls into question simple ideas about the relationship between positive thinking and the placebo effect as well as the assertion that people have “repeated successful medical experiences” [55]. We know too little about hope and more research is needed but the fact that it seems that patients are more comfortable talking about hope than expectation suggests that researchers may need to incorporate this psychological model into discussions of placebo effects.

### Uncertainty

Uncertainty involves “seeing into the future” but it is seldom thought to be associated with placebo responses. Nonetheless, we have two reasons to consider including uncertainty as one component of our patients’ complex responses. First, our previous qualitative studies of IBS patients found that patients expressed profound uncertainty about their future improvement (usually as part of their discussion of hope), and yet they still responded to placebo treatments. Second, there is a small body of prospective research that suggests that positive expectancies combined with some doubt may produce greater placebo responses than positive expectancy alone [59, 60]. Along these lines, electrophysiological brain research in monkeys suggests that dopamine, a neurotransmitter that plays a critical role in many placebo responses, is significantly increased under conditions of uncertainty [61]. Clearly, OLP is a condition of uncertainty for many patients; however, we do not yet know how exactly uncertainty, expectancy, and/or hope is related to the experience of OLP. Therefore, for rational and ethical reasons, we designed the current study to allow for a reasonably positive expectancy or hope for a positive outcome, and, at the same time our interactions purposefully allow for uncertainty by avoiding any implied promise of improvement. In fact, as we mentioned earlier, when patients express their uncertainty or feeling that the rationale makes no sense, we easily share their perspective. We acknowledge participants’ skepticism and our own but we express genuine hope and interest in the results.

### Classical conditioning

If the placebo effect can be elicited without conscious expectations, as discussed above, perhaps classical conditioning applies to our patients. A classical conditioning explanation of the placebo effect involves an associative learning process in which an unconditioned stimulus (the active drug) is paired with an initially neutral stimulus (the ritual of pill taking), and these stimuli are reliably followed by the unconditioned response (symptomatic improvement in response to the active medication). With a sufficient number of pairings, the neutral stimulus (the ritual of pill taking) becomes a conditioned stimulus, such that it can by itself produce symptomatic reductions which are termed conditioned responses. This theory, though, seems inadequate to fully explain placebo effects and especially the OLP effect. Most of the patients in the various OLP experiments cited in this paper had seen multiple clinicians before enrolling in the study and had long histories of treatment failure [37]. It seems unlikely that they were simply conditioned positively with previous experiences, except perhaps for the migraine experiment [18]. And indeed, one commentator has even suggested that novel therapies (which would include OLP) “may provide an opportunity for ‘deconditioning’ from previous unsuccessful medical experiences” [62].

### Prediction processing

Another model emerging from computational biology and cognitive science that some of our team members have tentatively explored, and which is not necessarily incompatible with the models above, is “prediction processing.” This model considers the brain as an organ of prediction (or a “prediction machine”) [63–65]. For example, if a person is walking in a forest notorious for dangerous snakes, sometimes when they glance at an ordinary stick, the brain will visually process a snake. This sort of quick nonconscious prediction is necessary for survival [64]. Analogously, when you visit a caring physician, all the doing, seeing, touching, feeling, and knowing tells your brain that you’re in a healing situation. In our OLP paradigm, everything points to healing except that our participants know that they are taking placebos. Perhaps a more conscious part of the brain is predicting that nothing will happen (“after all, I’m taking a placebo”), while another, perhaps less conscious part, is hopeful that something will happen – or perhaps it switches back and forth as something happens (spontaneous improvement) or does not happen (no improvement). Either way, there are deviations from predicted states (“prediction error”) and the brain can correct errors and modulate perceptions of symptoms via top-down mechanisms, as mentioned earlier [63]. If the entire ritual surrounding OLP points to a possibility of improvement and there is some normal variability of actual improvement, the brain can follow a *post-hoc ergo hoc* fallacy that indeed the OLP placebo may be working and even release helpful neurotransmitters to consolidate this fallacy into some kind of concrete reality.

This kind of prediction process is related to “embodied cognition” theories that bypass critical evaluations of consciousness. For example, think of attendance at a Shakespeare play: no matter how many times you watch Romeo and Juliet commit suicide, as long as the performance is evocative, you might shed a tear, feel goose bumps, and get dryness in the mouth. The drama becomes real and palpable even if you know it is fiction and you have seen it many times before. The body knows and reacts accordingly, despite rationality implying differently [51, 66].

### Novelty

As mentioned earlier, we have been struck by the fact that our patients experience taking OLP placebo as “incongruous,” “crazy,” and certainly a novel experience. We have no idea how this fits into the psychology of responding to OLP but it should be mentioned that this sort of reaction commonly exists. Furthermore, novelty-seeking itself has been implicated with dopamine release and other placebogenic mechanisms [67, 68]. For many of our previous patients, the trial has been a fun experience. Would it be too far-fetched to suggest that we are dealing with an adult version of what psychoanalyst Winnicott called a “transitional object” [69]? Could the pills be an equivalent of the child’s “teddy bear,” an intermediate object between a difficult external reality (separation/illness) and an inner reality of hope, uncertainty, and even despair?

### Other proposed psychological mechanisms

Other psychological explanations for response to OLP might include anxiety reduction, cognitive reappraisal, social learning, and selective attention, all of which have some empirical support [49, 67, 68]. Patients may also feel empowered by our discussion of “self-healing processes” and our explanation that placebo may activate an endogenous internal pharmacy.

### Patient-clinician relationship

OLP is generally given in the context of a warm, caring, and attentive clinical interaction. There is significant evidence that placebo effects are enhanced by such engagement [31]. It is possible that this alone causes improvement in symptoms although in earlier OLP studies, and in this one ongoing one, we spend equal time with OLP and NTC and try to keep the patient-clinician interactions similar in time and attention. Unfortunately, there are not yet any empirical data comparing a supportive therapeutic relationship alone to a supportive therapeutic relationship plus OLP. Until such data are available, we speculate in psychodynamic terms, that the pill physically embodies the trust and good feeling of the provider. Certainly, we observed in many of our previous OLP patients a sense of play.

### Clinical implications of OLP and future directions for research

How might OLP be valuable moving forward? Many patients with symptoms are routinely placed on medication for subjective complaints. We know that even without a placebo effect, regression to the mean and spontaneous improvement will render a certain percentage of patients better in a few weeks [70, 71]. Therefore, one potential future application of OLP in a clinical setting may be in enhancing the common “watch-and-wait” strategy used when patients present with subjective complaints that may, if given time, resolve on their own. These are the instances in which providers might find themselves considering the use of “impure” placebos in order to make the waiting time more palpable to patients, many of whom prefer a more active approach than simply waiting. Adding an OLP to this strategy might make the “watch-and-wait” approach more acceptable and, potentially, more successful by supplementing the chance of spontaneous improvement with the possibility of a placebo effect. This strategy has clear benefits, especially given that it would allow patients and providers to avoid starting new medications or increasing dosages of medications with potentially high adverse effects profiles. OLP could also be tried as a first line of treatment for common symptoms when patients are reluctant to start taking medications and are willing, or want, to try OLP.

### Patient and physician acceptance

One important issue to consider is whether patients or health care providers will be willing to try OLP. The answer for patients seems to be “yes” as documented in a large survey of patients’ attitudes about the use of placebo (*n* = 853) that was performed at a major hospital in the USA. When presented with an OLP vignette based on our first IBS study, nearly 85% of patients considered OLP acceptable in general. In a second, more specific vignette that specifically spoke of “patients with chronic abdominal pain” being offered OLP with a similar script to our current study, 65% of patients saw this as acceptable [72]. Similarly, a focus group performed in the UK (*n* = 58) found that patients were generally comfortable with OLP if provided by a physician [73]. To our knowledge, there are no data to characterize physicians’ opinions about OLP and it is not clear whether they would accept OLP as a therapeutic option. We suspect that there may be challenges to physician acceptance given that their professional identity is closely tied to a history of disqualifying therapies because they are “only a placebo effect” [9].

### Bioethics

Ultimately, OLP is fundamentally an attempt to ethically harness placebo effects. Is it ethical? The current American Medical Associates (AMA) guidelines on the clinical use of placebos clearly states that “physicians may use placebo for diagnosis or treatment only if the patient is informed and agrees to its use” [74]. OLP is consistent with these AMA guidelines in that it based on transparency, respect for the person, and full and accurate information disclosure. A recent bioethical analysis published in *Bioethics* stated that “open placebos fulfill current (AMA) guidelines for placebo use” [15]. If further scientific evidence continues to support OLP, it probably would be worthwhile to encourage OLP instead of the common deceptive use of “impure” placebos, where physicians give pharmaceuticals that they know will have no physiological effect on the condition.

### Trial status

The study began recruitment in 2016 and is currently enrolling participants.

## Additional file

Additional file 1: SPIRIT 2013 Checklist. (DOC 121 kb)

## Abbreviations

AMA: American Medical Association
cLBP: Chronic lower back pain
COMT: Catechol-*O*-methyltransferase
DBP: Double-blind placebo
fMRI: Functional magnetic resonance imaging
IBS: Irritable bowel syndrome
IBS-SSS: Irritable Bowel Syndrome Severity Scoring System
NTC: No-treatment control
OLP: Open-label placebo
RCT: Randomized controlled trial.
